# HIST3H2A is a potential biomarker for pancreatic cancer: A study based on TCGA data

**DOI:** 10.1097/MD.0000000000027598

**Published:** 2021-11-19

**Authors:** Mengyu Zhao, Rongyang Dai

**Affiliations:** Department of Biochemistry and Molecular Biology, Southwest Medical University, Luzhou, Sichuan Province, PR China.

**Keywords:** HIST3H2A, JAK-STAT pathway, pancreatic cancer, prognosis, therapeutic target

## Abstract

The family of histone H2A proved that there are a lot of variants associated with cancer development. The link between HIST3H2A and pancreatic cancer has never been noted before. Our research suggests that HIST3H2A affects pancreatic tumor immune process and prognosis of patients, through the JAK STAT pathway, so it is expected to become the biomarker of pancreatic cancer.

Gene expression profiles and clinical data of pancreatic cancer patients were downloaded from The Cancer Genome Atlas database (TCGA) and The Genotype Tissue Expression (GETx) project. R software (Rx64 3.6.0) was utilized to analyze. Gene set enrichment analysis (GSEA) was used to analyze HIST3H2A related signaling pathways in pancreatic cancer. CIBERSORT is applied to estimate the compositional patterns of the 22 types of immune cell fraction based on bulk expression data.

HIST3H2A was expressed at higher in pancreatic cancer tissues than normal pancreatic tissues. Kaplan–Meier survival analysis suggested that the level of HIST3H2A expression affect prognosis of pancreatic cancer patients. Univariate Cox analysis and Multivariate Cox analysis suggested that HIST3H2A expression is a prognostic factor of pancreatic cancer. Cor expression analysis indicated that the genes positively correlated with HIST3H2A expression trend were DCST1-AS1, HIST1H2BD, SLC12A9-AS1. GSEA showed that the JAK-STAT signaling pathway was enriched in the HIST3H2A high expression phenotype, whereas intestinal network for IgA production, Asthma and Chemokine signaling pathway were enriched in the HIST3H2A low expression phenotype. In additional, results showed that CD8 T cells (*P* = .007), activated CD4 memory T cells (*P* = .001), and monocytes (*P* = .002) were more abundant in lower HIST3H2A expression groups.

HIST3H2A is a promising biomarker for predicting prognosis of pancreatic cancer, and it could be a potential therapeutic target. HIST3H2A might regulate the progression of tumor immune in pancreatic cancer through modulating the JAK-STAT pathway. In addition, the role HIST3H2A in pancreatic cancer may be related to DCST1-AS1, HIST1H2B, SLC12A9-AS1. However, more research is necessary to validate findings.

## 1 Introduction

Pancreatic cancer is one of the most lethal malignant neoplasms across the world. According to the GLOBOCAN 2012 estimate, pancreatic cancer causes >331,000 deaths per year, which accounts for 4.0% of all deaths, making it the 7th leading cause of cancer deaths in both sexes. The risk of developing pancreatic cancer increased with age, as the general population continues to age, this incidence trend is expected to increase. The estimated 5-year survival rate for pancreatic cancer is less than about 5%.^[1–3]^ Presently, the main treatment of pancreatic cancer is surgery. On account of lacking feasible method of screening and specific symptoms at an early stage, the majority of patients detected at advanced stage and lost the opportunity of surgical therapy. Unfortunately, few effective chemotherapeutic options for metastatic pancreatic cancer. In addition, its standard treatment, gemcitabine, was not evaluated independently or in combination with other chemotherapy drugs to exert a positive effect on survival in patients with advanced disease. Therefore, it is urgent for us to focus on further understanding the genetic and molecular factors contributing to oncogenesis, and find effective biomarkers to increase the rate of early diagnosis, treat, and predict prognosis of pancreatic cancer, thus greatly prolonging the life of pancreatic patients.^[3]^

The nucleosome particle is the basic unit of the chromatin fiber, in eukaryotic cells, which core is composed of histones H2A, H2B, H3, and H4. Additionally, the 2 units in H3 and H4 form a tetramer surrounded by 2 dimers of H2A-H2B.^[4]^ Cells can send dynamic signals to chromatin through DNA methylation, histone variation, and covalent modification of histones, so as to regulate gene transcriptions, DNA repair, and maintain genome integrity.^[5–8]^ Histones come in many translated forms, listed as acetylation, methylation, ubiquitination, and phosphorylation. Those modifications regulate many signaling pathways, transcriptional activation or inhibition,^[9]^ DNA repair, chromosome dynamics.^[10]^ In eukaryotes, H2A, H2B, and H3 have been shown to have variants, some of which are common, highly conserved in eukaryotes, and some of which are particularly evolved in eukaryotes.^[11]^ Presently, some studies have linked some variants of H2A to cancer. H2A.Z is considered to be mainly pro-tumor, whereas macroH2A and H2A.X are considered to be tumor suppressors.^[12]^ The frequency of H2A.Z overexpression is associated with poor survival in melanoma,^[13]^ hepatocellular carcinoma,^[14]^ and breast cancer.^[15]^ MacroH2A has been shown to have tumor inhibition in bladder cancer, melanoma, and tumor tetratomic,^[16]^ and breast cancer.^[11]^ The deletion and mutation of H2A.X gene can cause severe DNA damage and has been associated with sporadic breast cancer,^[17]^ head and neck squamous cell carcinomas,^[18]^ neuroblastoma,^[19]^ and hematopoietic malignancies like chronic lymphocytic leukemia.^[11,20]^ s139 phosphorylated H2A.X, which is a promising marker for cancer risk assessment, early detection, prognosis, and treatment evaluation, can quantitatively detect DNA damage in cells and tissues. s139 phosphorylated H2A.X was shown to be associated with lung, breast, lung, ovarian, and colorectal cancer.^[21]^ Phosphorylated histone H2AX now is considered to be a biomarker of precancerous liver cancer.^[22]^ Although there have many studies on the relationship between histone variants and covalent modifications of the canonical H2A family and cancer in recent years, few studies have been conducted on its relationship with cancer due to the small variation of the canonical H2A sequence, and the functional significance of the H2A is still unclear at present.

In this work, we attempted to reveal the significance of HIST3H2A, in canonical family, expression in pancreatic cancer. The expression of HIST3H2A may affect the process of tumor immunity in pancreatic cancer, thus affecting the prognosis of pancreatic cancer patients. We compared HIST3H2A mRNA expression between tumor tissues and normal tissues and correlated them with clinical parameters, then we analyzed the correlation between those data and patients’ overall survival (OS) and disease-free survival (DFS). Additionally, we performed GSEA analysis and co-expression analysis to explore the specific molecular mechanism of this gene in pancreatic cancer. Finally, we applied a kind of deconvolution algorithm to reveal the proportions of 22 subsets of immune cells in pancreatic cancer.

## 2 Materials and methods

### 2.1 Database

The data included 178 pancreatic cancer tissues and 4 normal tissues, patient clinical information (include futime, fustat, age, sex, pathological stage, and histological grade) and the HIST3H2A expression levels were downloaded from The Cancer Genome Atlas (TCGA) database (https://genome-cancer.ucsc.edu). In addition, we collected 203 normal pancreatic tissues into TCGA database to perform variance analysis.

The data are downloaded from a public database and therefore do not apply to additional ethical approvals.

### 2.2 Statistical analysis

We perform all statistical analyses through R software (Rx64 3.6.0). First, we extract gene expression profiles and pancreatic cancer patient's data, then we annotated the gene matrix and delete patients with incomplete clinical information. Secondly, we divided the patients according to the expression of the HIST3H2A into 2 groups (high and low) and correct the expression and patients’ OS and DFS using the Kaplan–Meier method. Thirdly, we analyzed the correlation between patients’ survival state and clinical information via Wilcoxon rank sum test, Kruskal-Wallis test, then examined the role of each factor via logistic regression. Univariate Cox analysis was used to select possible prognostic factors, and multivariate Cox analysis was utilized to verify the correlations between HIST3H2A expression and survival along. Using the survival ROC package we create a receiver-operating characteristic curve to evaluate the accuracy of the models.

### 2.3 Gene set enrichment analysis

We figured out which pathway the gene set related to HIST3H2A expression were enriched in through GSEA, and analyzed the possible intermolecular interaction mechanism through co-expression analysis. Gene set permutations were performed 1000 times for each analysis. Gene sets with a *P* value <.05 and false discovery rate <0.05 were regarded as significantly enriched.

### 2.4 Evaluation of immune cell subtype distribution

To quantify the relative proportions of infiltrating immune cells from the gene expression profiles in pancreatic cancer, a bioinformatics algorithm called CIBERSORT (https://cibersortx.stanford.edu/) was used to calculate immune cell infiltrations. The putative abundance of immune cells was estimated using a reference set with 22 types of immune cell subtypes with 1000 permutations. Violin plots were drawn using the“vioplot” package in R to visualize the differences in immune cell infiltration between the higher HIST3H2A expression and lower HIST3H2A expression.

## 3 Result

### 3.1 Clinical parameters of patients

The clinical data of pancreatic cancer patients were obtained from the TCGA database and included age, sex, clinical stage, classification, vital state, among others (Table 1).

**Table 1:** Clinicopathological characteristics of patient samples and expression of HIST3H2A in pancreatic cancer.

Characteristics	No. of cases (%)
Age, y	
≥65	93 (55.3)
<65	75 (44.7)
Sex	
Male	92 (54.8)
Female	76 (45.2)
Clinical stage	
I	18 (10.7)
II	143 (85.1)
III	3 (1.79)
IV	4 (2.41)
T classifcation	
T1	5 (2.98)
T2	23 (13.7)
T3	137 (81.5)
T4	3 (1.82)
N classifcation	
N0	47 (28.0)
N1	121 (72.0)
Metastasis	
M0	77 (45.8)
M1	4 (2.4)
M2	87 (51.8)
Vital states	
alive	83 (49.4)
Dead	85 (50.6)
Expression of HIST3H2A	
Low expression	83 (49.4)
High expression	85 (50.6)

### 3.2 HIST3H2A is highly expressed in pancreatic cancer

**Figure 1:**
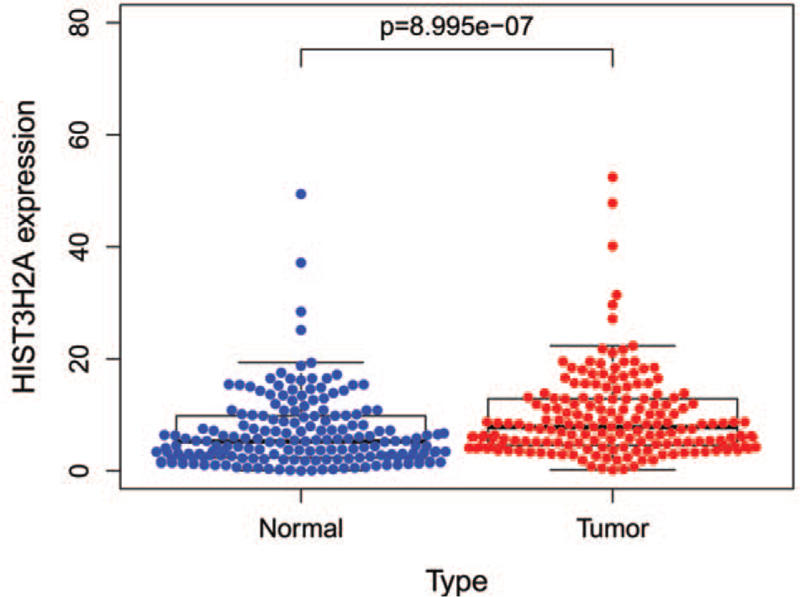
The scatter plot showed the difference of HIST3H2A expression between normal and tumor samples.

Via Wilcoxon rank sum test, we compared 178 pancreatic cancer tissues and 207 normal tissues. The result shows that HIST3H2A expression is significantly high in pancreatic tissues (Fig. 1; *P* < .001).

### 3.3 Correlations between HIST3H2A expression and clinical parameters in pancreatic cancer patients

We analyzed the relationship between HisT3H2A and patients’ clinical parameters by R software (Rx64 3.6.0). The Logistic regression analysis result indicated that the increase of HIST3H2A expression in pancreatic cancer patients was not correlated with these clinical parameters (age, sex, clinical stage, T stage N stage and M stage, vital state) (Table 2).

**Table 2:** Correlation between HIST2H2A expression and clinicopathologic characteristics of pancreatic cancer patients.

	HIST3H2A		
Characteristics	Low expression	High expression	*P*
Age, y			
≥65	40	41	.797
<65	47	40	
Sex			
Male	45	47	.679
Female	42	34	
Clinical stage			
I	11	7	.779
II	73	70	
III	2	1	
IV	1	3	
T classifcation			
T1	3	2	.451
T2	14	9	
T3	68	69	
T4	2	1	
N classifcation			
N0	23	24	.77
N1	64	57	
Metastasis			
M0	43	34	.476
M1	1	3	
M2	43	44	
Vital states			
Alive	49	34	.119
Dead	38	46	

### 3.4 HIST3H2A could be an independent risk factor for predicting pancreatic cancer prognosis

**Figure 2:**
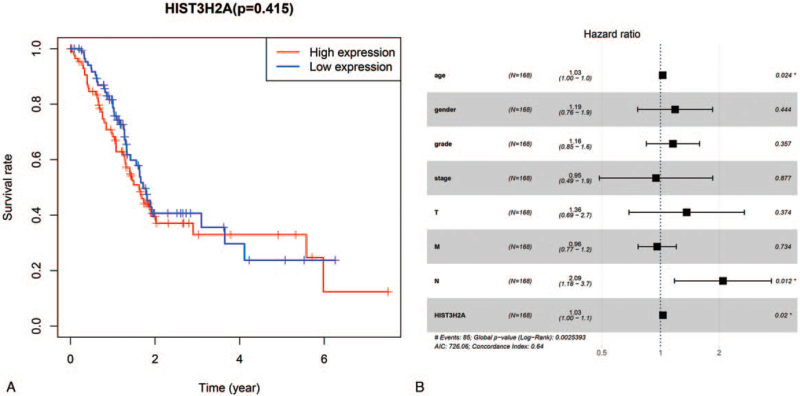
(A) Survival analysis demonstrated that PC with HIST3H2A-low had a more terrible prognosis than that with HIST3H2A-high; (B) Cox regression analysis of HIST3H2A expression as a tumor survival predictor.

We analyzed the relative risks indicated by HIST3H2A in the prognosis of pancreatic cancer. All pancreatic cancer patients were categorized according to the median HIST3H2A expression value (high HIST3H2A expression group and low HIST3H2A expression group). We excluded the patient without complete clinical data. Kaplan-Meier survival analysis has no significance (Fig. 2A). The univariate Cox regression analysis indicates that high expression of HIST3H2A was associated with a significantly increased risk of death in pancreatic cancer patients (*P* < .05) compared to those with low HIST3H2A expression (Table 3). Some clinical parameters, such as age (*P* < .05), N classification (*P* < .05) also correlated with prognosis of pancreatic cancer (Table 4). Multivariate Cox regression analysis indicated that HIST3H2A could be an independent risk factor for predicting disease prognosis (*P* < .05), and age (*P* < .05), histological grade (*P* < .05), N classification (*P* < 0.05) were included (Table 2 and Fig. 2B).

**Table 3:** Cox regression analysis of HIST3H2A expression as a tumor recurrence predictor.

	Univariate Cox regression analysis		Multivariate Cox regression analysis	
Variables	HR (95% CI)	*P*	HR (95% CI)	*P*
Age	1.028 (1.005–1.051)	.016	1.026 (1.003–1.049)	.024
Sex	1.280 (0.834–1.965)	.259	1.189 (0.763–1.851)	.444
Grade	1.331 (0.981–1.806)	.066	1.159 (0.847–1.585)	.007
Stage	1.294 (0.862–1.942)	.214	0.948 (0.485–1.853)	.877
T	1.624 (0.977–2.701)	.062	1.362 (0.689–2.693)	.374
M	0.961 (0.772–1.196)	.724	0.962 (0.768–1.205)	.734
N	2.258 (1.308–3.898)	.003	2.091 (1.179–3.708)	.012
HIST3H2A	1.025 (1.002–1.048)	.034	1.029 (1.004–1.055)	.020

CI = confidence interval, HR = hazard ratio.

**Table 4:** Results of GSEA gene enrichment.

Gene set name	NES	*P*	FDR-value
KEGG Glycine Serine and Threonine metabolism	−0.66	.037	0
KEGG Intestinal Immune Network for IGA Production	−0.74	.044	0.011
KEGG Asthma	−0.74	.046	0.014
KEGG Chemokine Signaling Pathway	−0.55	.047	0.018
KEGG_JAK_STAT_Signaling_Pathway	0.53	.049	0.002

GSEA = Gene set enrichment analysis.

### 3.5 Cor expression analysis of the expression of HIST3H2A associated pancreatic genome

**Figure 3:**
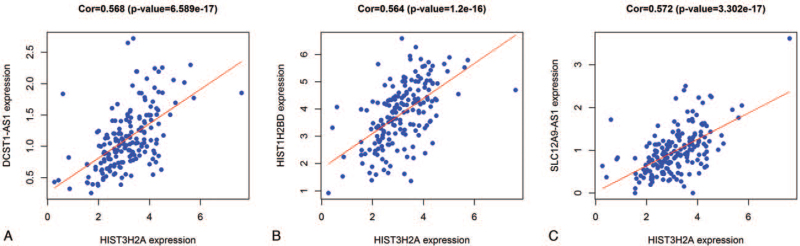
Cor expression analysis of the expression of HIST3H2A associated pancreatic genome.

To study the genes associated with HIST3H2A expression in pancreatic cancer, we performed cor expression analysis. The results showed that the genes positively correlated with HIST3H2A expression trend were DCST1-AS1 (*P* < .001) (Fig. 3A), HIST1H2BD (*P* < .001) (Fig. 3B), SLC12A9-AS1 (*P* < .001) (Fig. 3C).

### 3.6 JAK -STAT signaling pathway and intestinal immune network are regulated by HIST3H2A

**Figure 4:**
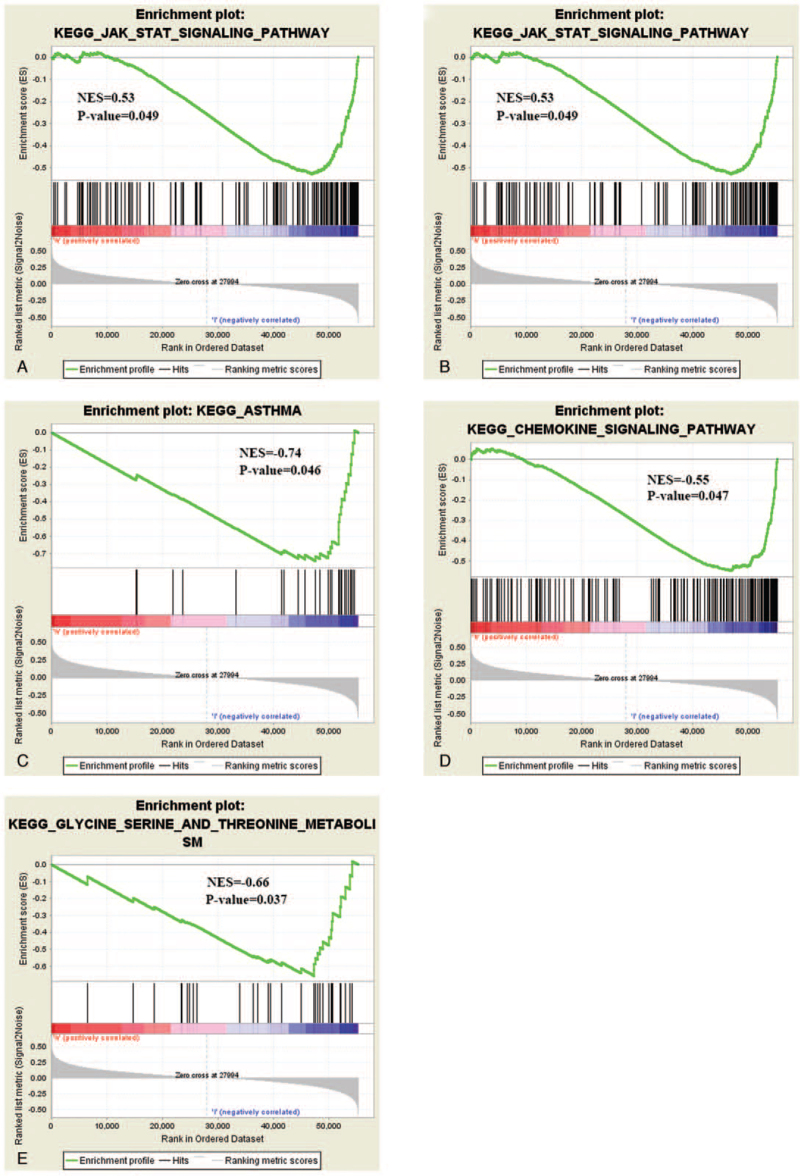
Enrichment plots from gene set enrichment analysis (GSEA).

To further study the regulatory pathway of HIST3H2A in pancreatic cancer, we performed a GSEA analysis (Table 4) in the published TCGA pancreatic cancer database. We found that the higher expression of HIST3H2A was positively correlated with the JAK-STAT signaling pathway (Fig. 4A), whereas intestinal immune network for IgA production (Fig. 4B), asthma (Fig. 4C), chemokine signaling pathway (Fig. 4D), glycine serine, and threonine metabolism (Fig. 4E) were enriched in the HIST3H2A low expression phenotype, suggesting that pathway may be involved in the function of HIST3H2A.

### 3.7 The landscape of immune cells infiltration in pancreatic cancer

**Figure 5:**
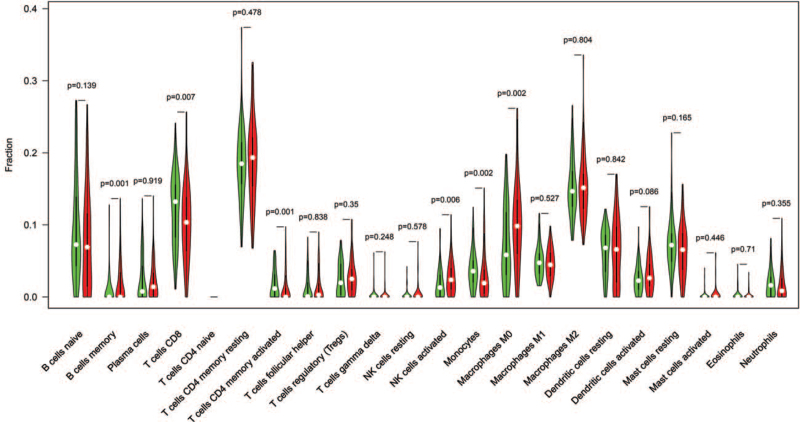
Visualization of immune cell infiltration, comparison of 22 immune cell subtypes between lower HIST3H2A and higher HIST3H2A expression groups. Green and red colors represent lower HIST3H2A and higher HIST3H2A expression groups, respectively.

To further study the tumor immune function of HIST3H2A **in pancreatic cancer**, we used the CIBERSORT algorithm to estimate the TCGA-**pancreatic cancer** immune cell infiltration status and compared the differences in immune cells infiltration patterns between higher HIST3H2A and lower HIST3H2A (Fig. 5). The results showed that Lower HIST3H2A expression. The results showed that CD8 T cells (*P* = .007), activated CD4 memory T cells (*P* = .001), and monocytes (*P* = .002) were more abundant in lower HIST3H2A expression groups. Although memory B cells (*P* = .001), activated NK cells (*P* = .006), and M0 macrophages (*P* = .002) were significantly higher in higher HIST3H2A expression groups. This finding indicates that immune-activated cells were significantly richer in the lower HIST3H2A expression, which also consolidates the result of GSEA in the low HIST3H2A expression phenotype.

## 4 Discussion

The JAK STAT pathway mediates the action of cytokines, interferons, and growth factors, and control of gene expression in cell. It transduces extracellular signals into transcriptional programs which regulate cell growth and differentiation, and it also has assumed additional functions, participating in the regulation of chromatin conformation and epigenetic marking in the nuclei and affecting oxidative phosphorylation, in the cytoplasm, the mitochondria, and the nucleus.^[23]^ It is worth noting that, the JAK STAT pathway plays an important role in the immune system. The mutations of JAKs and STATs can cause immune deficiencies. It results in the occurrence of nonfunctional T cells and defective immunoglobulin producing cells, which impedes the development of T cells, NK cells, and the function of B cells. STAT3 and STAT5 activity is required for the generation of human T follicular helper cells. (T follicular helper cells can induce B cells to differentiate and produce antibodies.)^[24]^ The JAK STAT pathway is activated by interleukin (IL)6, IL10/IL22, and IL17/IL23 cytokine family members and participates in intestinal immune. Over-activated the JAK pathway inhibits tumor immune function in innate and adaptive immune, thus promoting the growth of malignant cells.^[25]^ Our study showed that higher expression of HIST3H2A was positively correlated with the JAK-STAT signaling pathway. In addition, by analyzing immune cell infiltration in tumor microenvironment, we found that immune-activated cells (CD8 T cells and memory activated CD4+ T cells) were significantly insufficient in the higher HIST3H2A expression. Thus, we speculate that HIST3H2A might regulate the progression of tumor immune in pancreatic cancer through modulating the JAK-STAT pathway. In HIST3H2A survival curve, the 3 years preceding the trend is very clear, but by the late trend is no longer obvious, we think that pancreatic cancer patients tend to develop rapidly, and patients who survived for >3 years may have stronger tumor immune function due to genetic factors, so they were less affected by HIST3H2A.

High expression of LncRNA DST1-AS1 was significantly correlated with inferior prognosis. LncRNA DCST1-AS1 promoted cell proliferation and invasion, inhibited apoptosis and autophagy by modulating the AKT/mTOR signaling cascade.^[26]^ High expression of the HIST1H2BD was significantly correlated with the prognosis of cervical cancer patients.^[27]^ H2A/H2B plays important roles in processes on the chromatin that allow for transcription, DNA replication, and DNA repair. The SLC12 gene family encodes electroneutral inorganic cation chloride cotransporters, which are plasma membrane proteins mediating the movement of inorganic sodium (Na+)and potassium(k+) cations, tightly coupled to the movement of chloride (cl+) anions. These transporters play several important roles in human physiology. The SLC12 family of proteins includes SLC12A8 and SLC12A9, which encode proteins for which no function has yet been ascribed.^[28]^ Due to the limited research on HIST3H2A in pancreatic cancer patients, So the interaction between HIST3HI2A and these genes (SLC12A8 and SLC12A9) needs further experimental study. We demonstrated the association of these genes using bioinformatics methods to further influence the development and prognosis of pancreatic cancer patients by influencing the course of tumor immunity.

That said, there were also a few limitations to this study. First, we only analyzed TCGA database and GETx project, our results were needed to be validated in many external validation cohorts; the batch effects from different cohorts should be considered to confirm these intriguing findings. Second, we did not determine the optimal cutoff value of HIST3H2A. Here, the median Siglec15 mRNA expression was considered as the cutoff value. In our study, Kaplan-Meier survival analysis has no significance. However, the univariate Cox regression analysis indicates that high expression of HIST3H2A was associated with a significantly increased risk of death in pancreatic cancer patients compared to those with low HIST3H2A expression. Multivariate Cox regression analysis also indicated that HIST3H2A could be an independent risk factor for predicting disease prognosis. Finally, further experiments are needed to determine the relationship between the expression profiles of HIST3H2A, JAK-STAT signaling pathway, and immune cell infiltration.

## 5 Conclusions

HIST3H2A is a promising biomarker for predicting prognosis of pancreatic cancer, and it could be a potential therapeutic target. HIST3H2A might regulate the progression of tumor immune in pancreatic cancer through modulating the JAK STAT pathway. In addition, the role of HIST3H2A in pancreatic cancer may be related to DCST1-AS1, HIST1H2B, SLC12A9-AS1. However, more research is necessary to validate findings.

## Author contributions

MYZ and YZZ conceived and designed the study. MYZ and YZZ drafted the manuscript. MYZ and YZZ analyzed and interpreted all the data. MYZ and YZZ prepared the figures and tables. MYZ and YZZ reviewed and revised the

manuscript. All authors have read and approved the manuscript for publication.

**Conceptualization:** Mengyu Zhao, Rongyang Dai.

**Data curation:** Mengyu Zhao, Rongyang Dai.

**Formal analysis:** Mengyu Zhao, Rongyang Dai.

**Funding acquisition:** Mengyu Zhao, Rongyang Dai.

**Investigation:** Mengyu Zhao, Rongyang Dai.

**Methodology:** Mengyu Zhao, Rongyang Dai.

**Project administration:** Mengyu Zhao, Rongyang Dai.

**Resources:** Mengyu Zhao, Rongyang Dai.

**Software:** Mengyu Zhao, Rongyang Dai.

**Supervision:** Rongyang Dai.

**Validation:** Rongyang Dai.

**Visualization:** Rongyang Dai.

**Writing – original draft:** Rongyang Dai.

**Writing – review & editing:** Rongyang Dai.
